# Chimpanzee Adenovirus Antibodies in Humans, Sub-Saharan Africa

**DOI:** 10.3201/eid1210.060078

**Published:** 2006-10

**Authors:** Zhiquan Xiang, Yan Li, Ann Cun, Wei Yang, Susan Ellenberg, William M. Switzer, Marcia L. Kalish, Hildegund C.J. Ertl

**Affiliations:** * The Wistar Institute, Philadelphia, Pennsylvania, USA;; † University of Pennsylvania School of Medicine, Philadelphia, Pennsylvania, USA;; ‡ Centers for Disease Control and Prevention, Atlanta, Georgia, USA

**Keywords:** Neutralizing antibodies, prevalence, chimpanzee adenovirus, chimpanzee-to-human transmission, Africa, vaccine, HIV-1, dispatch

## Abstract

Human sera from the United States, Thailand, and sub-Saharan Africa and chimpanzee sera were tested for neutralizing antibodies to 3 chimpanzee adenoviruses. Antibodies were more common in humans residing in sub-Saharan Africa than in humans living in the United States or Thailand. This finding suggests cross-species transmission of chimpanzee adenoviruses.

Vaccines to HIV-1 are needed to stem further spread of the HIV pandemic. One vaccine modality that has shown promise is based on adenovirus vectors of human serotype 5 (AdHu5) (*1**–**3*). However, immune responses induced by AdHu5 vectors are reduced by preexisting AdHu5-neutralizing antibodies found in humans in the United States (*4**–**7*).

## The Study

To circumvent the negative effect of preexisting immunity to common human serotypes of adenoviruses on the efficacy of adenovirus vaccine carriers, we developed vectors based on chimpanzee-derived adenoviruses C68, C6, and C1 (*8*). We previously showed that neutralizing antibodies to chimpanzee adenoviruses are rarely found in US residents (*6*). Because vaccines to HIV-1 are most urgently needed in sub-Saharan Africa, we evaluated the prevalence of neutralizing antibodies to chimpanzee adenoviruses in sera from humans residing in 3 sub-Saharan countries with natural habitats for chimpanzees: Nigeria, Cameroon, and Côte d'Ivoire. Sera from captive chimpanzees in the United States and human sera from Thailand and the United States, including samples from persons with known exposures to chimpanzees, were tested for comparison.

Neutralizing antibodies to AdHu5 were found in most serum samples from Africa and Thailand. Percentages of antibodies to AdHu5 were higher in serum samples from Africa and Thailand than in serum samples from the United States (Table 1). Titers to AdHu5 were comparable in serum samples from the United States and sub-Saharan Africa but were higher in the control group from Thailand (Table 2).

**Table 1:** Sera with neutralizing activity to different human and chimpanzee adenoviruses

Origin	% positive samples (p values)*†			
	AdHu5	AdC68	AdC6	AdC1
Human controls, United States (n = 50)	34.0	2.0	4.0	2.0
Human zoo workers, United States (n = 50)	28.0	0	0	0
Humans, Thailand (n = 200)	76.5	1.5	3.0	4.0
Humans, Cameroon (n = 405)	55.8	1.7 (0.6764)	7.9 (0.0045)	5.4 (0.1248)
Humans, Côte d'Ivoire (n = 169)	95.8	9.5 (0.0003)	10.7 (0.0008)	3.0 (0.9796)
Humans, Nigeria (n = 182)	89.0	4.9 (0.0267)	18.7 (<0.0001)	9.3 (0.0045)
Chimpanzees, United States (n = 50)	44.0	86.0 (<0.0001)	92.0 (<0.0001)	46.0 (<0.0001)

*p values show statistical difference between percentages of sera positive for neutralizing antibodies to human and chimpanzee adenoviruses. Reactivity of human sera from Cameroon, Côte d'Ivoire, and Nigeria and of chimpanzee sera to the 3 chimpanzee-derived adenoviruses were compared with human sera from the United States (n = 100) and Thailand (n = 200); the last 2 were combined because these countries do not offer natural chimpanzee habitats (similarity of USA and Thailand data for these adenoviruses was statistically confirmed). A logistic regression model was fitted to compare the percentages of samples positive for neutralizing antibodies between different groups. A p value <0.05 was considered statistically significant. All analyses were performed by using SAS version 9.1 logistic procedure (*9*). †Virus tested for neutralization with a previously described neutralization assay (*10*). Samples that neutralized virus at dilutions >1:20 were scored as positive.

**Table 2:** Mean adenovirus neutralizing antibody titers for positive samples

Origin	Mean VNA* titer ± standard deviation†			
	AdHu5	AdC68	AdC6	AdC1
Humans, United States	**116 ± 111**	40 ± 0	20 ± 0	20 ± 0
Humans, Thailand	**303 ± 353‡**	20 ± 0	80 ± 44	**33 ± 25**
Humans, Cameroon	**125 ± 114**	109 ± 145	**82 ± 52‡**	**58 ± 41**
Humans, Côte d'Ivoire	**162 ± 212**	**60 ± 51‡**	**37 ± 34‡**	148 ± 275
Humans, Nigeria	**165 ± 206**	29 ± 20	**80 ± 115‡**	**42 ± 23**
Chimpanzees, United States	**164 ± 233**	**201 ± 204**	**137 ± 160**	**64 ± 72**

*VNA , virus neutralizing antibody. †p values were determined by a Student *t* test to assess differences between titers in experimental samples and control samples. Human sera from the United States were used as a reference for antibodies to AdHu5, and chimpanzee sera were used as a reference for titers to AdC68, AdC6, and AdC1. Experimental samples in which >10 samples were positive for the given adenovirus are shown in **boldface**. ‡Samples that showed a statistically significant difference (p<0.05).

Neutralizing antibodies to AdC68, AdC6, and AdC1 were rare in sera from the United States and Thailand, with prevalence rates from 1.5% to 4% (Figure). Most positive samples had titers <80. Of 200 samples from Thailand, only 6 had high titers: 4 to AdC6 and 2 to AdC1. None of the samples from 50 US zoo workers, including those who reported regular contact with primates, had detectable antibodies to chimpanzee adenoviruses.

**Figure:**
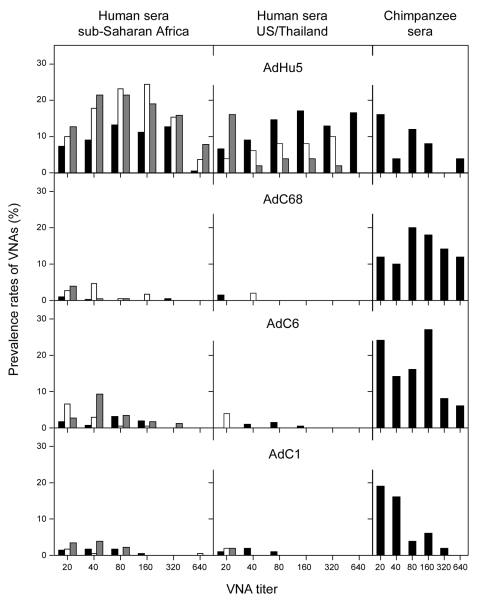
Prevalence of neutralizing antibody titers to chimpanzee adenoviruses. Percentages of negative samples are not shown. Left column: Cameroon, black bars; Côte d'Ivoire, white bars; Nigeria: gray bars. Middle column: Thailand, black bars; US controls, white bars; US zoo keepers or animal handlers, gray bars. VNAs, virus neutralizing antibodies. Coded human serum samples that had been collected for other studies were obtained under an institutional review board exemption.

In contrast, serum samples from sub-Saharan African cohorts had higher prevalences of neutralizing antibodies to chimpanzee adenoviruses (Figure, Table 1). Samples from persons living in Cameroon and Nigeria showed a higher prevalence of neutralizing antibodies to AdC6 and AdC1. Titers of neutralizing antibodies to AdC6 and AdC1 were high, and samples were positive at dilutions >1:80. When compared with sera from Nigeria and Cameroon, sera from Côte d'Ivoire had a different pattern of antibodies reactive to the chimpanzee adenoviruses; prevalence rates were low to AdC1 but higher to AdC68 and AdC6. Although most samples had low to moderate titers, several samples had titers >80. No association was found between neutralizing antibodies to AdC68, AdC6, and AdC1 in any of the human samples tested, and only a few human serum samples had neutralizing antibodies to >1 chimpanzee adenovirus (data not shown). One serum sample from Cameroon neutralized all 3 chimpanzee adenoviruses and had titers of 20 to AdC68 and 160 to AdC6 and AdC1.

Circulating neutralizing antibodies to AdHu5 were found in 44% of captive US chimpanzees; titers in chimpanzee samples were comparable to those in human sera, suggesting that AdHu5 can readily cause an infection in captive chimpanzees. The prevalence of antibodies to AdC6 and AdC68 was high and exceeded that of antibodies to AdC1 (Table 1). Titers to AdC68 and AdC6 were higher in chimpanzees than in humans, but titers to AdC1 in both species were similar (Table 2).

## Conclusions

Our data show that as expected, neutralizing antibodies to chimpanzee adenoviruses are rarely found in humans residing in the United States or Thailand. In contrast, their prevalence is higher in human sera from sub-Saharan Africa, where hunting and butchering of nonhuman primates for food are widespread and eating bush meat is common (*11*). Different prevalence rates of neutralizing antibodies to the 3 chimpanzee adenoviruses in human samples from the 3 African countries tested may reflect different infection rates in chimpanzees residing in these areas.

Cameroon, Gabon, and Republic of Congo are home to most Central African common chimpanzees (*Pan troglodytes troglodytes*). Most Western common chimpanzees (*P*. *t*. *verus*) inhabit Côte d'Ivoire and Guinea. The rare Nigeria chimpanzee (*P*. *t*. *vellerosus*) is found only in eastern Nigeria and western Cameroon, and Eastern African common chimpanzees (*P*. *t*. *schweinfurthii*) reside in a range from Central African Republic and the Democratic Republic of the Congo through western Uganda and Tanzania. No clear association was found between chimpanzee subspecies and neutralizing antibodies to different chimpanzee adenoviruses. Nevertheless, most samples were from *P*. *t*. *verus*, and only a limited number of samples were from *P*. *t. troglodytes* (n = 4) and *P*. *t*. *schweinfurthii* (n = 3). No samples from *P*. *t*. *vellerosus* were tested. In addition, the chimpanzee samples evaluated in the study were from animals kept in captivity and thus may not provide information on distribution of these viruses in free-ranging animals.

Increased prevalence of neutralizing antibodies to chimpanzee adenoviruses in sub-Saharan Africa may reflect cross-species transmission of these viruses from chimpanzees to humans. If transmission occurs, human-to-human spread of chimpanzee adenoviruses might further contribute to the comparatively high seroprevalence seen in equatorial Africa. Although we have no direct proof for viral cross-species transmission, this process has been previously described for other chimpanzee viruses. For example, the AIDS epidemic is believed to have originated from simian immunodeficiency virus–infected chimpanzees (*12*). Another chimpanzee retrovirus, simian foamy virus, has been reported to infect persons exposed to primates at zoos and research centers (*13*) and to infect Bantus in Cameroon (*14*). Chimpanzee adenoviruses do not appear to spread easily to humans through occupational contact with primates because none of the 23 persons who had routine exposure to primates, including chimpanzees, had serologic evidence of exposure despite the high prevalence of antibodies to chimpanzee adenoviruses in captive US chimpanzees.

Use of chimpanzee adenovirus vectors as vaccines for HIV-1 would require that most persons at high risk for HIV-1 infection lack neutralizing antibodies to these adenoviruses because such antibodies impair induction of transgene product-specific immune responses (*4**,**5*). Even in countries where chimpanzees are endemic, neutralizing antibodies to chimpanzee adenoviruses are less common in humans than those directed to AdHu5, which is currently in clinical trials as a vaccine for HIV-1 antigens in a vectored and replication-defective form. An alternative human serotype is AdHu35, which is being developed as a potential vaccine against HIV-1 (*15*). Prevalence rates to AdHu35 are low in the United States and Europe (<5%); however, rates are markedly higher (<20%) in equatorial Africa (*15*).

Although neutralizing antibodies to chimpanzee adenoviruses are also relatively more common in human sera from sub-Saharan Africa, they are found less frequently than antibodies to AdHu5 or AdHu35. Nonetheless, increased prevalence of neutralizing antibodies to adenoviruses in countries that are hardest hit by the AIDS pandemic needs to be taken into account in the design of vaccines based on chimpanzee adenovirus vectors. Vectors derived from other species are being developed and may provide additional or alternative vaccine carriers.
